# A preclinical study of deep brain stimulation in the ventral tegmental area for alleviating positive psychotic-like behaviors in mice

**DOI:** 10.3389/fnhum.2022.945912

**Published:** 2022-08-10

**Authors:** Chen Lu, Yifan Feng, Hongxia Li, Zilong Gao, Xiaona Zhu, Ji Hu

**Affiliations:** ^1^School of Life Science and Technology, Shanghai Tech University, Shanghai, China; ^2^Institute of Neuroscience, State Key Laboratory of Neuroscience, CAS Center for Excellence in Brain Science and Intelligence Technology, Shanghai Institutes for Biological Sciences, Chinese Academy of Sciences, Shanghai, China; ^3^University of Chinese Academy of Sciences, Beijing, China; ^4^Department of Neurology and Institute of Neurology, Ruijin Hospital Affiliated to Shanghai Jiao Tong University School of Medicine, Shanghai, China; ^5^School of Life Sciences, Westlake University, Hangzhou, China

**Keywords:** deep brain stimulation, psychosis, ventral tegmental area, GABA neurons, preclinical study

## Abstract

Deep brain stimulation (DBS) is a clinical intervention for the treatment of movement disorders. It has also been applied to the treatment of psychiatric disorders such as depression, anorexia nervosa, obsessive-compulsive disorder, and schizophrenia. Psychiatric disorders including schizophrenia, bipolar disorder, and major depression can lead to psychosis, which can cause patients to lose touch with reality. The ventral tegmental area (VTA), located near the midline of the midbrain, is an important region involved in psychosis. However, the clinical application of electrical stimulation of the VTA to treat psychotic diseases has been limited, and related mechanisms have not been thoroughly studied. In the present study, hyperlocomotion and stereotyped behaviors of the mice were employed to mimic and evaluate the positive-psychotic-like behaviors. We attempted to treat positive psychotic-like behaviors by electrically stimulating the VTA in mice and exploring the neural mechanisms behind behavioral effects. Local field potential recording and *in vivo* fiber photometry to observe the behavioral effects and changes in neural activities caused by DBS in the VTA of mice. Optogenetic techniques were used to verify the neural mechanisms underlying the behavioral effects induced by DBS. Our results showed that electrical stimulation of the VTA activates local gamma-aminobutyric acid (GABA) neurons, and dopamine (DA) neurons, reduces hyperlocomotion, and relieves stereotyped behaviors induced by MK-801 (dizocilpine) injection. The results of optogenetic manipulation showed that the activation of the VTA GABA neurons, but not DA neurons, is involved in the alleviation of hyperlocomotion and stereotyped behaviors. We visualized changes in the activity of specific types in specific brain areas induced by DBS, and explored the neural mechanism of DBS in alleviating positive psychotic-like behaviors. This preclinical study not only proposes new technical means of exploring the mechanism of DBS, but also provides experimental justification for the clinical treatment of psychotic diseases by electrical stimulation of the VTA.

## Introduction

The clinical application of deep brain stimulation (DBS), a neurosurgical procedure commonly used to treat movement disorders such as Parkinson’s disease, tremor, and dystonia, has been one of the most important advances in clinical neuroscience in the last two decades (Montgomery and Baker, 2000; Udupa and Chen, 2015; Pycroft et al., 2018). DBS is also increasingly being used to treat psychiatric disorders such as obsessive-compulsive disorder, depression, anorexia nervosa, and schizophrenia (Kopell et al., 2004; Kuhn et al., 2010; Holtzheimer and Mayberg, 2011; Graat et al., 2017), but the neural mechanism underlying the effects of treatment remains unclear, which has limited the extension of DBS to other brain regions.

Psychosis is an abnormal condition of the mind that results in losing contact with reality. It can be caused by schizophrenia, bipolar disorder, psychotic depression (Sachdev, 1998; Keck et al., 2003; Craddock et al., 2005; Read et al., 2005; DeBattista et al., 2006; Schatzberg et al., 2014), trauma, sleep deprivation, and drugs such as cannabis and methamphetamine (Meyer and Meyer, 2009; Leboyer et al., 2012; Jones et al., 2014; Waters et al., 2018). The main symptoms include hallucinations and delusions (Haddock et al., 1999; Morrison, 2001; Freeman and Garety, 2003; Garety et al., 2005; Schultze-Lutter et al., 2012). The preferred treatment for psychosis is antipsychotic medication (Seeman, 2002; Davis et al., 2003; Lieberman et al., 2005). However, due to poor target-specificity, antipsychotics can cause many metabolic side effects, such as obesity, hyperlipidemia, and hyperglycemia (Alvarez-Jimenez et al., 2008; Patel et al., 2009). Owing to its excellent specificity, DBS has been gradually applied in the treatment of clinical psychiatric disorders (Kopell et al., 2004). Previous studies have reported that local electrical stimulation of the nucleus accumbens (NAc), the lateral habenula (LHb), and the anterior cingulate cortex (ACC) in patients diagnosed with schizophrenia can alleviate the positive and cognitive symptoms (Kuhn et al., 2011; Ma and Leung, 2014; Nucifora et al., 2019; Corripio et al., 2020; Roldán et al., 2020; Wang et al., 2020; Germann et al., 2021). Additional potentially relevant brain areas remain to be explored.

The ventral tegmental area (VTA) is an important brain region involved in the onset and etiology of psychosis (D’Ardenne et al., 2008; Cohen et al., 2012; Lammel et al., 2012; Morales and Margolis, 2017). According to clinical imaging data, compared to healthy individuals, the activity of the VTA in patients with psychiatric disorders is significantly lower (Lisman et al., 2010; Hadley et al., 2014; Rausch et al., 2014; Rice et al., 2016; Yamashita et al., 2016; Giordano et al., 2018; Sotoyama et al., 2021). Therefore, in clinical studies, this area has also been included among the candidate brain regions for the treatment of psychiatric disorders by electrical stimulation (Georgiev et al., 2021). For example, stimulating the VTA in patients can effectively relieve the frequency and severity of headaches in patients with chronic headache (Miller et al., 2016; Akram et al., 2017; Vyas et al., 2019). Gazit et al. (2015) found that DBS of the VTA reduced depression-like behaviors in rats. However, there are limited clinical and experimental studies using electrical stimulation in the VTA in the treatment of psychotic symptoms. Therefore, in the present study, we attempted to treat positive psychotic-like behaviors by electrically stimulating the VTA in mice and illustrating the precise working mechanism.

## Materials and Methods

### Animals

The care and use of animals were conducted in strict accordance with institutional guidelines and governmental regulations. All mice were maintained under a reversed 12-h /12-h day/night cycle at 22–25°C with *ad libitum* access to rodent food and water in environmentally controlled conditions. The mice used in the experiments were adult (8–15 weeks) C57BL/6 male mice (Shanghai Model Organisms), *Vgat-ires-cre* knock-in mice (Stock No. 028862) and *DAT-ires-cre* knock-in mice (Stock No. 006660; Jackson Laboratory, Bar Harbor, ME). The engineered mice were both maintained on a C57BL/6J genetic background.

All experiments involving mice were carried out in accordance with the US National Institutes of Health Guide for the Care and Use of Animals under an Institutional Animal Care and Use Committee approved protocol and Association for Assessment and Accreditation of Laboratory Animal Care approved Facility at the ShanghaiTech University.

### Viral injection, fiber optics, and DBS electrode implantation

After the mice were deeply anesthetized with isoflurane, they were placed on a stereoscopic positioning instrument. The eyes were coated with aureomycin eye cream, and the scalp was cut open. Then, the skull surface was wiped with 3% hydrogen peroxide to remove the fascia from the skull surface. The Bregma point and Lambda point were used to adjust the mouse head to the horizontal position. A small window with a diameter of 300–500 microns was opened at the location of viral injection and fiber implantation. According to the brain atlas, the ML range of the VTA is ±0.2–1.2 mm. Since both electrical stimulation and illumination stimulation act on the neurons within a certain range, we chose ±0.5 and ±0.7 mm, which are closer to the center, as the experimental coordinates. The viral injection rate was 300 nl/min. AAV2/9-hsyn-DIO-GCaMP6(m) was injected unilaterally for fiber photometry [Anterior–Posterior (AP), −3.10 mm; Medial–Lateral (ML), ±0.5 mm; and Dorsal-Ventral (DV), −4.25 mm, Fiber tip: −4.10]. rAAV-EF1a-DIO-hChR2(H134R)-mCherry/rAAV-EF1a-DIO-eNpHR3.0-mCherry was injected bilaterally for optogenetic experiments [Anterior–Posterior (AP), −3.10 mm; Medial–Lateral (ML), ±1.15 mm; and Dorsal-Ventral (DV), −4.25 mm, Fiber tip: −4.06]. For bilateral viral injection in the VTA, the syringe was angled 8° laterally to avoid the central sinus. After the viral injection, the apparatus remained in place for at least 10 min. Then, the fiber (200-micron inner diameter, NA = 0.37), the DBS electrodes (Coated Platinum-Iridium Wire, 76.2-micron inner diameter, Cat No. 777000, A-M system, USA), and the *in vivo* LFP electrodes (PFA-Coated Stainless Steel Wire, 75-micron, Cat No. 791000, A-M system, USA) were slowly implanted into the VTA unilaterally or bilaterally [Anterior–Posterior (AP), −3.10 mm; Medial–Lateral (ML), ±0.5 mm; and Dorsal-Ventral (DV), −4.00 mm]. Then the optical fiber, DBS electrodes, *in vivo* LFP electrodes, and skull were fixed with dental cement. After the dental cement was completely dried, the mice were removed from the positioning instrument and placed on an electric blanket. After the mice fully recovered, they were put back into the feeding cage.

### Fiber photometry recording

GCaMp6m expressed in VTA DA and GABA neurons using *DAT-ires-cre* and *Vgat-ires-cre* mice through intra-VTA injection of AAV2/9-hsyn-DIO-GCaMP6m. Following AAV-DIO-GCaMP6m viral injection, an optical fiber (200 μm outer diameter, 0.37 numerical aperture; Anilab) was placed in a ceramic ferrule and inserted toward the VTA through the craniotomy. Mice were individually housed for at least 2 weeks to recover. Fluorescence signals were acquired with a fiber photometry system equipped with a 488 nm excitation laser, 505–544 nm emission filter, and a photomultiplier tube (R3896, Hamamatsu). The analog voltage signals were digitized at 100 Hz and recorded by a Power 1401 digitizer and Spike2 software (CED, Cambridge, UK). An optical fiber (RJPSF2, Thorlabs) with an integrated rotary joint preventing fiber damage from the animal movement was used to guide the light between the fiber photometry system and the implanted optical fiber. The laser power was adjusted at the tip of the optical fiber to the low level of 20–40 μW, to minimize bleaching.

For the fiber photometry experiments, the analysis methods were as follows. Photometry data were exported to MATLAB R2020b mat files from Spike2 for further analysis. We segmented the data based on behavioral events within individual trials. The event time was recorded manually at every point of the stimulation. We derived the values of fluorescence change (ΔF/F) by calculating (F − F_0_)/F_0_, where F_0_ is the baseline fluorescence signal averaged over a 5 s-long control time window. ΔF/F values were presented with average plots to illustrate the signal changes trial by trial. To calculate the average response and decrease the duration of ΔF/F values, we first segmented the data based on the behavioral events and baseline phase. Then we calculated the average calcium signal both in baseline and event phases.

### The evaluation of positive psychotic-like behaviors in mice

Hyperlocomotion and stereotyped behaviors are thought to evaluate positive psychotic symptoms in mice (Eichler et al., 1980; Angermeyer and Matschinger, 2004; Morrens et al., 2006; van den Buuse, 2010; Forrest et al., 2014; Schubart et al., 2014; Compton et al., 2015; Svoboda et al., 2015; Kaufmann et al., 2018; Ma and Guest, 2018; Dahlén et al., 2021).

Locomotion was obtained through the open field test. Animals were placed in the center zone of a 40 × 40 × 40 cm open field chamber in a room with dim light. A video camera positioned directly above the chamber was used to record the movement of each test mouse. An automated video-tracking system was controlled by MATLAB R2018b. The total distance traveled during the session was tracked and further analyzed.

Stereotyped behaviors were rated off-line by a trained observer who was blind to the treatment. Criteria for scoring the intensity of stereotyped behaviors mainly followed a previous study (Sams-Dodd, 1996) with some slight modifications, which are as follows. (0): Immobility, little or no movement. (1): Normal activity and occasional forward movement. (2): Activity accompanied by repeated exploration. (3): Continuous forward exploration. (4): Repeatedly raising and shaking the head or spinning the body. (5): A quick shake of the head, circling, or dorso-ventral movements of the head (usually while standing still). The score assigned for each behavioral category was determined as the highest level of stereotyped behaviors consistently observed during the rating period, which time ratio is over 50%.

During the open field test, mice were first injected intraperitoneally with 0.3 mg/kg MK-801. Thirty minutes later, local electrical stimulation was started at 60 μs, 130 Hz, and 100 μA for 1 min. The analysis indexes, hyperlocomotion, and stereotyped behaviors of the mice were recorded for 1 min before, during, and after electrical stimulation respectively.

### Optogenetic light delivery and protocols

*Vgat-ires-cre* mice were used for the experiments. rAAV-EF1a-DIO-hChR2(H134R)-mCherry and rAAV-EF1a-DIO-eNpHR3.0-mCherry were injected into the VTA. Two optical fibers were implanted above the VTA. After 3 weeks of virus expression, the experiments were carried out. First, the mice were allowed to adapt to the open field for 10 min. For mice expressing eNpHR3.0, a 555-nm yellow light laser was delivered continuously for 180 s. Light intensity was calculated to be about 10 mW. The interval between the two stimulations was 10–15 min. For mice expressing ChR2, first, we injected 0.3 mg/kg MK-801 and then placed the animal in the open field environment. Twenty minutes later, we began to activate the neurons with a 473-nm blue light laser delivered at 20 Hz in 15 ms pulses for 180 s. Light intensity was calculated to be about 10 mW. The interval between the two stimulations was 10–15 min.

### DBS stimulation delivery and protocols

DBS stimulation: electrical current (60 μs, 100 μA, 130 Hz).

Electrical stimulator: STG4002, Germany.

In our study, the current polarity is bipolar stimulation. There are two electrodes on each side of the VTA, which are connected to the positive and negative poles of the stimulator respectively. The metal electrode at 0.5 mm of the tip of the electrode is exposed and the rest is covered by an insulating layer. One electrode serves as the cathode while another serves as the anode.

### *In vivo* recording of LFPs

Mice were unilaterally implanted with a 75-μm stainless-steel electrode (Cat No. 791000, A-M system, USA) in the VTA (AP: −3.10 mm from Bregma, ML: ± 0.7 mm from the midline, DV: −4.0 mm from meninx). They were anesthetized with isoflurane for the implantation procedure (3% for induction 3%, 1.5% for maintenance) and allowed to recover for 1 week after surgery. Recording signals (low-pass filter: 1–100 Hz) were digitized by the Ephyslab System (Thinker Tech Nanjing Biotech Co. Ltd.) at 30 kHz, and then resampled at 1 kHz for the LFP analysis.

During the recording process, LFPs of the VTA neurons were recorded under normal conditions as a baseline value. MK-801 (0.3 mg/kg), which takes 30 min to exert its effects, was intraperitoneally injected into the mice as a pharmacological model of psychosis. The LFPs of the VTA in the last 5 min of the 30 min post-administration period were recorded as the pathogenic values under pathological conditions. Next, the VTA was stimulated with a 60 μs wave width, 130 Hz, and 100 μA current for 1 min. After the stimulation stopped, LFPs were recorded for 5 min as the therapeutic value. Finally, MATLAB R2020b was used for statistical analysis. Target oscillations (gamma: 25–100 Hz, Delta: 1–4 Hz, Theta: 4–8 Hz, Alpha: 8–12 Hz, Beta: 12–25 Hz) were classified based on previous studies.

The videos were recorded simultaneously with a camera and the LFP data were analyzed using MATLAB R2018b. The recorded LFPs were filtered by a 50 Hz notching filter to remove the powerline artifact. The target oscillations (gamma: 25–100 Hz, Delta: 1–4 Hz, Theta: 4–8 Hz, Alpha: 8–12 Hz, Beta: 12–25 Hz) were divided according to previous studies. Raw data were transformed by Spike2 software (CED, Cambridge, UK) initially, then analyzed by using MATLAB R2018b. In order to avoid current disturbance due to DBS stimulation, the headstage of the LFP was removed from the mice as soon as the stimulation was over. The normalized power was defined as the power value, with every point divided by the mean of the baseline (before MK-801) groups.

### Immunohistochemistry

To verify the expression of adeno-associated viral functional proteins, we performed immunohistochemistry of tyrosine hydroxylase in the VTA. The mice were deeply anesthetized through the abdominal cavity with pentobarbital (100 mg/kg), and then saline was perfused through the heart. After most blood was drained out, 4% paraformaldehyde (PFA) was used for fixation. To better fix the brain tissue, the head was removed and soaked in 4% PFA at room temperature overnight. The brain was removed the next day, post-fixed overnight in 4% PFA at 4°C, and transferred to 30% sucrose in 0.1 M PBS, pH 7.4 for 24–48 h. Coronal sections (20 μm) containing the VTA were cut on a cryostat (Leica CM3050S). The slides were washed with 0.1 M PBS, pH 7.4, incubated in blocking buffer (0.3% Triton X-100, 5% bovine serum albumin in 0.1 M PBS, pH 7.4) for an hour, and then with primary antibodies (rabbit anti-tyrosine hydroxylase antibody, 1:1,000; Invitrogen) in blocking buffer overnight at 4°C. After washing three times with 0.1 M PBS, pH 7.4, the sections were incubated with donkey anti-rabbit IgG H&L (fluor-488 or fluor-594; 1:1,000; Abcam) secondary antibody at room temperature for 2 h. DAPI (4’,6-diamidine-2-phenindoles) staining was used to identify the cell bodies. Finally, 10% glycerin was used to seal the slides. Fluorescent images were collected using a confocal microscope (Zeiss 800).

## Quantification and Statistical Analysis

### Data analysis

The data from brain sections were processed with ImageJ software. All statistical analyses were performed with GraphPad Prism 7.0 or MATLAB R2018b. The type of statistical analysis in our study is one-way ANOVA. Data were considered statistically significant when *p* < 0.05, “ns” means no significance. Asterisks denote statistical significance (*p < 0.05; **p < 0.01; ***p < 0.001; and ****p < 0.0001). No statistical methods were used to predetermine the sample size. Unless otherwise indicated, values are reported as the mean ± SEM.

The list of key resources is detailed in Table 1.

**Table 1:** Key resources table.

**Reagent or resource**	**Source**	**Identifier**
**Antibodies**		
Rabbit polyclonal anti-TH	Invitrogen	Cat# p21962
**Bacterial and Virus Strains**		
AAV2/9-hsyn-DIO-GCaMP6m	Taitool Bioscience	N/A
AAV2/9-EF1a-DIO-mCherry	Taitool Bioscience	N/A
rAAV-EF1a-DIO-hChR2(H134R)-mCherry	BrainVTA	N/A
rAAV-EF1a-DIO-eNpHR3.0-mCherry	BrainVTA	N/A
**Chemicals, Peptides, and Recombinant Proteins**		
**(+)-MK 801 (Dizocilpine)**	TOCRIS	Cat. No. 0924
**Experimental Models: Organisms/Strains**		
*DAT-ires-cre*	Jackson Laboratory	Stock No. 006660
*Vgat-ires-cre*	Jackson Laboratory	Stock No. 028862
C57BL/6 mice	Shanghai Model Organisms	N/A
**Software and Algorithms**		
MATLAB R2018b	MathWorks	www.mathworks.com/
GraphPad Prism 7	GraphPad Software	www.graphpad.com/
FIJI (ImageJ)	NIH	https://imagej.nih.gov/ij/

## Results

### DBS of the VTA alleviates hyperlocomotion and stereotyped behaviors caused by MK-801 injection in mice

**Figure 1:**
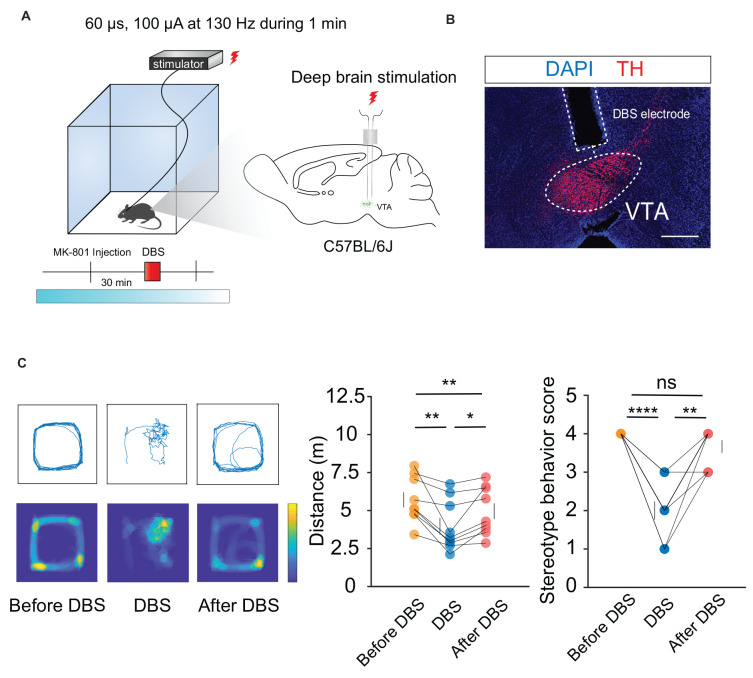
DBS of the VTA alleviates hyperlocomotion and stereotyped behaviors in mice. **(A)** Schematic of the DBS equipment and the DBS electrode location. **(B)** White dashed lines indicate the location of the DBS electrode above the VTA. Scale bar, 300 μm. TH, tyrosine hydroxylase. **(C)** The locomotor activity was quantified in an open field, in the presence or absence of DBS after intraperitoneal injection of MK-801. Deep brain stimulation of the VTA could decrease the hyperlocomotion and stereotyped behaviors caused by the MK-801 injection. *p < 0.05, **p < 0.01, ****p < 0.0001, ns: no significance. One-way ANOVA. All error bars represent ±SEM. *N* = 9 mice.

Hyperlocomotion and stereotyped behaviors, which are recognized as positive symptoms in animal models of psychosis, were chosen as the measurement indicators. Mice were first injected intraperitoneally with 0.3 mg/kg MK-801. Thirty minutes later, local electrical stimulation was started at 60 μs, 130 Hz, and 100 μA for 1 min (Figures 1A,B). The behavioral results showed that compared to the condition before and after the stimulation, hyperlocomotion and stereotyped behaviors of the mice recorded in 1 min were significantly reduced during electrical stimulation (Figure 1C). This part of the work showed that local electrical stimulation of the VTA can alleviate MK-801-induced positive psychotic-like behaviors in mice, which lays a foundation for the clinical application of this treatment in the future.

### DBS of the VTA reverses the local LFPs caused by MK-801 injection

**Figure 2:**
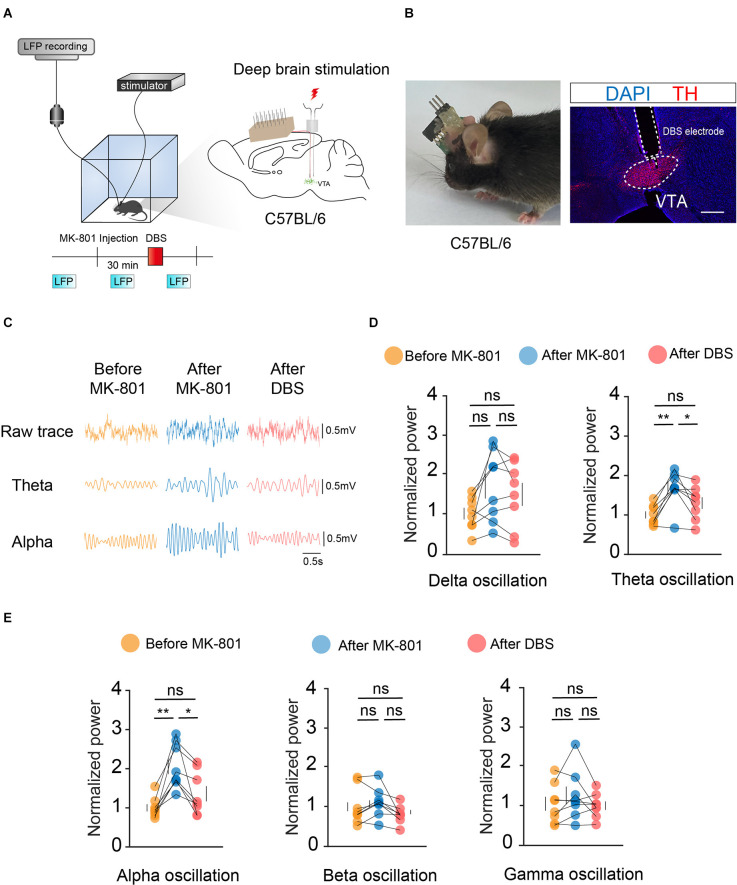
DBS of the VTA reverses the local LFPs caused by MK-801 injection. **(A)** Left: schematic of the DBS equipment and the field potential recording system. Right: schematic of *in vivo* electrophysiological electrode and DBS electrode implantation. **(B)** Left: actual view of the two electrodes on the head of a mouse. Right: white dashed lines indicate the location of the DBS electrode and electrophysiological electrode above the VTA. Scale bar, 300 μm. TH, tyrosine hydroxylase. **(C)** Example LFP trace of the mouse before MK-801 injection (i.p.), after MK-801 injection, and after DBS stimulation. **(D,E)** Quantification of average normalized Delta, Theta, Alpha, Beta, and Gamma band power in the VTA. Theta and alpha oscillation showed significant differences between the MK-801 injection and DBS stimulation. For all figures: one-way ANOVA with Tukey’s multiple comparisons test, *p < 0.05; **p < 0.01; ns, no significance. *N* = 8 mice. All error bars represent ±SEM.

To observe changes in the VTA before and after electrical stimulation, we first used Local Field Potential (LFP) recordings to record changes in VTA population neuronal activity. Bilateral symmetrical DBS and LFP electrodes were implanted into the VTA, and the mice were assessed after 2 weeks of recovery (Figures 2A,B).

Target oscillations (gamma: 25–100 Hz, Delta: 1–4 Hz, Theta: 4–8 Hz, Alpha: 8–12 Hz, Beta: 12–25 Hz) were classified based on previous studies. Statistical results showed significant differences in theta- and alpha-frequency waves before and after electrical stimulation of the VTA (Figures 2C–E). Theta- and alpha-frequency waves have been extensively studied in the context of motor control, and the present study further illustrated the important role of the VTA in motor gating. These results indicated that local electrical stimulation of the VTA can reverse the effects of MK-801 on the electrical activity of VTA neurons.

### DBS of the VTA activates VTA DA and GABA neurons

To verify the effects of DBS on VTA neurons, we used *in vivo* fiber photometry to observe the Ca^2+^ activity of VTA neurons in real-time. Because the VTA contains 60%–65% DA neurons, 30%–35% GABA neurons, and 2%–3% glutamate (Glu) neurons, we selected DA and GABA neurons for further study.

**Figure 3:**
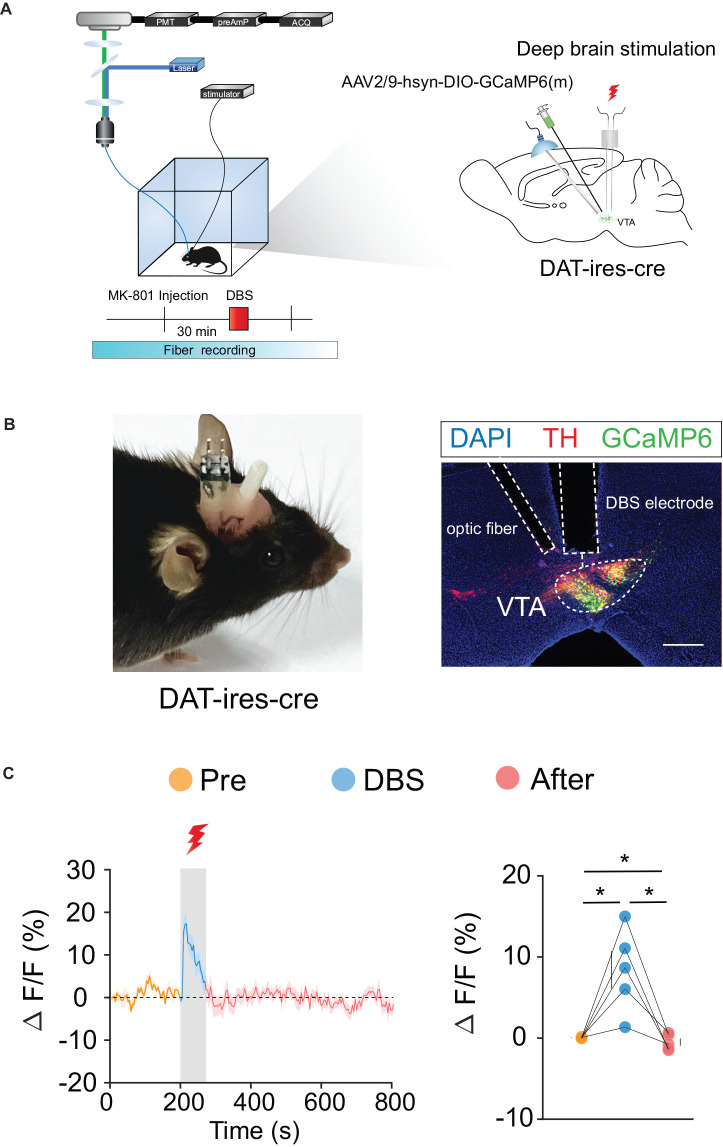
DBS of the VTA activates VTA DA neurons. **(A)** Left: schematic of the DBS equipment and the fiber photometry system. Right: schematic of viral injection, fiber implantation, and DBS electrode implantation in *DAT-ires-cre* mice. **(B)** Left: actual view of the electrode and the fiber on the head of a *DAT-ires-cre* mouse. Right: successful expression of GCaMP6m in VTA DA neurons. White dashed lines indicate the location of the DBS electrode and fiber above the VTA. The image shows GCaMP6m+ cell bodies (green) and TH+ neurons (red) in *DAT-ires-cre* mice. Scale bar, 300 μm. TH, tyrosine hydroxylase. **(C)** GCaMP6m signals from VTA DA neurons show that DBS of the VTA activates VTA DA neurons. *p < 0.05, one-way ANOVA. All error bars represent ±SEM. *n* = 5 mice.

**Figure 4:**
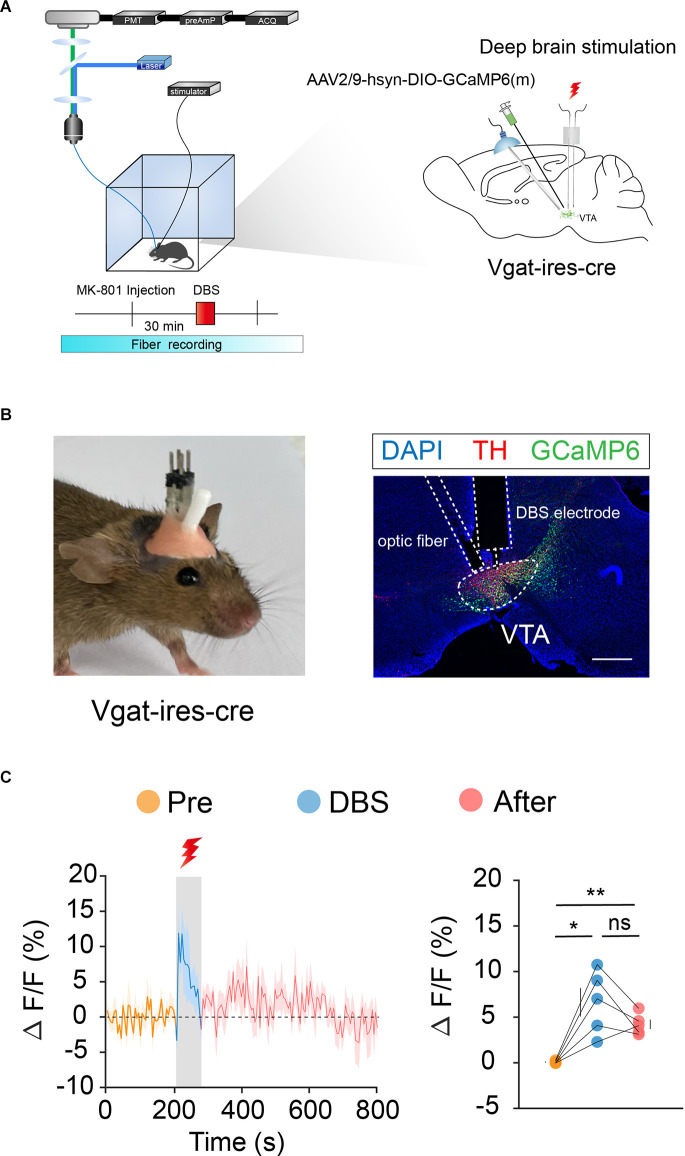
DBS of the VTA activates VTA GABA neurons. **(A)** Left: schematic of the DBS equipment and the fiber photometry system. Right: schematic of viral injection, fiber implantation, and DBS electrode implantation in *Vgat-ires-cre* mice. **(B)** Left: actual view of the electrode and the fiber on the head of a *Vgat-ires-cre* mouse. Right: successful expression of GCaMP6m in VTA GABA neurons. White dashed lines indicate the location of the DBS electrode and fiber above the VTA. The image shows GCaMP6m+ cell bodies (green) and TH+ neurons (red) in *Vgat-ires-cre* mice. Scale bar, 300 μm. TH, tyrosine hydroxylase. **(C)** GCaMP6m signals from VTA GABA neurons show that DBS of the VTA activates VTA GABA neurons immediately and they remain active for a period of time after the stimulus has stopped. *p < 0.05, **p < 0.01; ns, no significance. one-way ANOVA. All error bars represent ±SEM. *n* = 5 mice.

We labeled GCaMp6m in VTA DA (Figures 3A,B) and GABA (Figures 4A,B) neurons through intra-VTA injection of AAV2/9-hsyn-DIO-GCaMP6m in *DAT-ires-cre* and *Vgat-ires-cre* mice, respectively. MK-801 was intraperitoneally injected into mice as a pharmacological model of psychosis. The fiber recording results showed that the VTA GABA and DA neurons were rapidly activated when the electricity was turned on. Thus, these neurons showed continuous activation during electrical stimulation (Figures 3C and 4C). These findings further describe the effects of DBS on VTA local neurons from another perspective.

### Bidirectional regulation of VTA GABA neurons, rather than DA neurons, modulates positive psychotic-like behaviors

**Figure 5:**
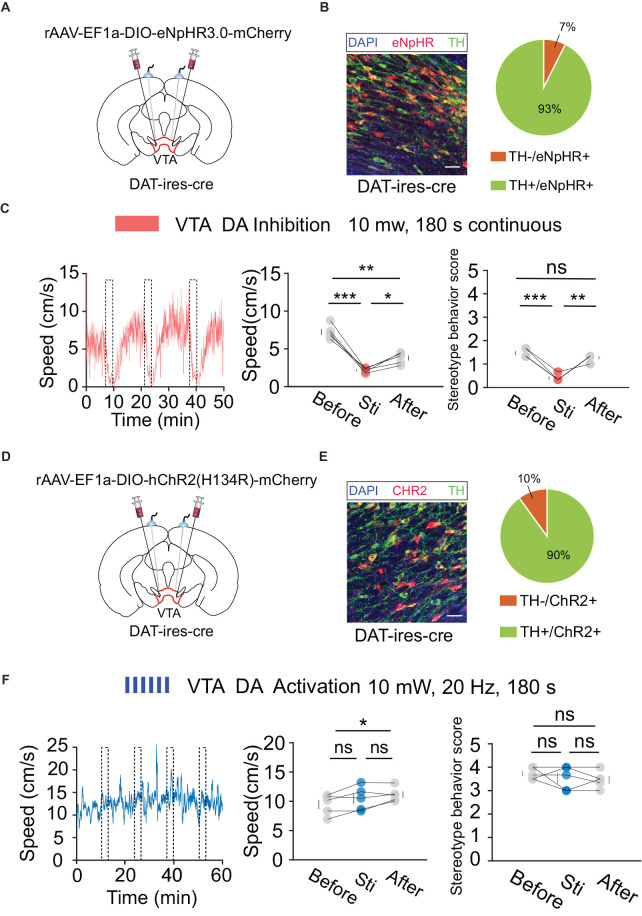
Optogenetic manipulation of VTA DA neurons. **(A)** Schematic of virus injection and fiber implantation above the VTA in *DAT-ires-cre* mice. **(B)** Successful expression of eNpHR3.0-mCherry in VTA DA neurons and optical fiber implantation above the VTA. The image shows eNpHR + cell bodies (red) and TH+ neurons (green) in *DAT-ires-cre* mice. Scale bar, 20 μm. TH, tyrosine hydroxylase. Right: quantification of expression of TH in eNpHR-positive neurons in the VTA of *DAT-ires-cre* mice (*n* = 3 sections per animal from five animals). **(C)** Left: the movement speed in response to 180 s photo inhibition of VTA DA neurons in *DAT-ires-cre* mice; middle: quantification of movement speed before, during, and after VTA DA photoinhibition. *p < 0.05, **p < 0. 01, ***p < 0.001; ns, no significance. one-way ANOVA. All error bars represent ±SEM. *n* = 5 mice; right: quantification of the stereotyped behavior scores before, during, and after VTA DA photoinhibition. **p < 0.01, ^***^p < 0.001, one-way ANOVA. All error bars represent ±SEM. *n* = 5 mice. **(D)** Schematic of viral injection and fiber implantation above the VTA in *DAT-ires-cre* mice. **(E)** Successful expression of hChR2 (H134R)-mCherry in VTA DA neurons and optical fiber implantation above the VTA. The image shows hChR2 + cell bodies (red) and TH+ neurons (green) in *DAT-ires-cre* mice. Scale bar, 20 μm. TH, tyrosine hydroxylase. Right: quantification (pie chart) of expression of TH in hChR2-positive neurons in the VTA of *DAT-ires-cre* mice (*n* = 3 sections per animal from five animals). **(F)** Left: the movement speed in response to 10 mW, 20 Hz, 180 s photoactivation of VTA DA neurons in *DAT-ires-cre* mice after intraperitoneal injection of MK-801. Middle: quantification of the movement speed before, during, and after VTA DA photoactivation. *p < 0.05, one-way ANOVA. All error bars represent ± SEM. *n* = 5 mice; right: quantification of the stereotyped behavior scores before, during, and after VTA DA photoactivation. One-way ANOVA. All error bars represent ±SEM, *n* = 5 mice.

According to the behavioral and fiber recording results described above, activation of VTA neurons mediated by DBS plays a crucial role in the regulation of hyperlocomotion and stereotyped behaviors in mice. However, the VTA primarily contains two types of neurons: DA and GABA. The type of neuron that plays a major role in this process remains to be explored. To verify the behavioral functions of DA and GABA neurons, we used optogenetic technology to analyze their respective roles. First, we injected the cre-dependent virus, rAAV-EF1a-DIO-eNpHR3.0-mCherry, into the VTA and implanted a fiber above the VTA of *DAT-ires-cre* mice (Figures 5A,B). After 3 weeks of expression, optogenetic inhibition and OFT behavioral tests were performed. The results showed that the optogenetic inhibition of VTA DA neurons significantly reduced locomotion in mice (Figure 5C). Similarly, we injected the cre-dependent virus rAAV-EF1a-DIO-hChR2(H134R)-mCherry into the VTA and implanted a fiber above the VTA of *DAT-ires-cre* mice (Figures 5D,E). After 3 weeks of expression, the mice were injected intraperitoneally with MK-801 (0.3 mg/kg), and 30 min later, optogenetic activation and OFT behavioral tests were conducted. The results showed that optogenetic activation of VTA DA neurons did not significantly affect hyperlocomotion and stereotyped behaviors in mice caused by MK-801 injection (Figure 5F).

**Figure 6:**
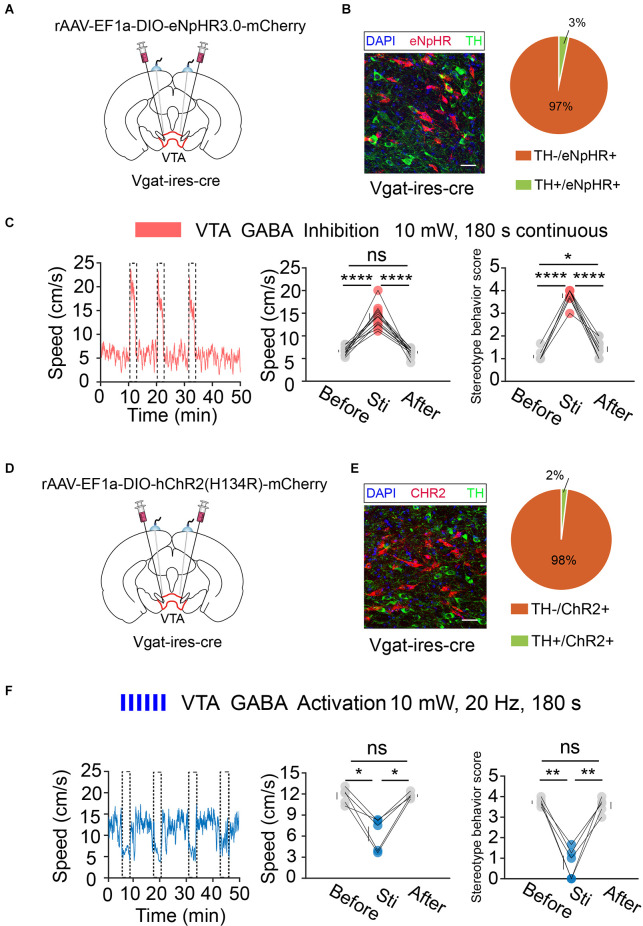
Optogenetic manipulation of VTA GABA neurons. **(A)** Schematic of viral injection and fiber implantation above the VTA in *Vgat-ires-cre* mice. **(B)** Successful expression of eNpHR3.0-mCherry in VTA GABA neurons and optical fiber implantation above the VTA. The image shows eNpHR + cell bodies (red) and TH+ neurons (green) in *Vgat-ires-cre* mice. Scale bar, 20 μm. TH, tyrosine hydroxylase. Right: quantification (the pie chart) of expression of TH in eNpHR-positive neurons in the VTA of *Vgat-ires-cre* mice (*n* = 3 sections per animal from five animals). **(C)** Left: the movement speed in response to 180 s photoinhibition of VTA GABA neurons in *Vgat-ires-cre* mice; middle: quantification of movement speed before, during, and after VTA GABA photoinhibition. ****p < 0.0001, one-way ANOVA. All error bars represent ±SEM. *n* = 12 mice; right: quantification of the stereotyped behavior scores before, during, and after VTA GABA photoinhibition. *p < 0.05, ****p < 0.0001, one-way ANOVA. All error bars represent ±SEM.*n* = 12 mice. **(D)** Schematic of viral injection and fiber implantation above the VTA in *Vgat-ires-cre* mice. **(E)** Successful expression of hChR2-mCherry in VTA GABA neurons and optical fiber implantation above the VTA. The image shows hChR2 + cell bodies (red) and TH+ neurons (green) in *Vgat-ires-cre* mice. Scale bar, 20 μm. TH, tyrosine hydroxylase. Right: quantification (pie chart) of expression of TH in hChR2-positive neurons in the VTA of *Vgat-ires-cre* mice (*n* = 3 sections per animal from five animals). **(F)** Left: the movement speed in response to 10 mW, 20 Hz 180 s photoactivation of VTA GABA neurons in *Vgat-ires-cre* mice after intraperitoneal injection of MK-801. Middle: quantification of the movement speed before, during, and after VTA GABA photoactivation. *p < 0.05, one-way ANOVA. All error bars represent ±SEM. *n* = 5 mice; right: quantification of the stereotyped behavior scores before, during, and after VTA GABA photoactivation. **p < 0.01, one-way ANOVA. All error bars represent ±SEM, *n* = 5 mice.

Next, we performed the same optogenetic procedure in *Vgat-ires-cre* mice (Figures 6A,B,D,E). The results showed that optogenetic inhibition of VTA GABA neurons significantly increased hyperlocomotion and stereotyped behaviors in mice, while optogenetic activation of the VTA GABA neurons could effectively reverse the hyperlocomotion and stereotyped behaviors induced by MK-801 (Figures 6C,F). These results indicated that VTA GABA neurons, rather than DA neurons, play a crucial role in the modulation of positive psychotic-like behaviors. Combined with the previous behavioral results and recording results of DBS in the VTA, we concluded that activation of VTA GABA neurons is the DBS target for alleviating positive psychotic-like behaviors caused by MK-801.

## Discussion

By utilizing DBS, local field potential recording, and *in vivo* fiber recording, we found that electrical stimulation of the VTA can relieve positive psychotic-like behaviors caused by MK-801 injection. Fiber recording and optogenetic results showed that the activation of VTA GABA neurons, rather than DA neurons, is involved in the cessation of psychotic behaviors mediated by DBS in the VTA.

Local electrical stimulation of the VTA can reduce hyperlocomotion and stereotyped behaviors in mice (Figure 1), suggesting that the VTA can be used as a candidate brain region for the treatment of positive symptoms of psychosis. Recently, researchers used 10 electrodes to map the brain activity of patients with major depression. They implanted a nerve stimulation device that triggered tiny electrical pulses in the ventral striatum region when high activity levels were detected in the amygdala. After a period of treatment, the depressive symptoms were effectively controlled (Scangos et al., 2021). Such a strategy can also be imitated in studies on DBS treatment for psychotic symptoms. According to clinical imaging data, the activity of the VTA and its connectivity to other areas in patients with psychiatric disorders are significantly lower than that in healthy controls (Lisman et al., 2010; Hadley et al., 2014; Rausch et al., 2014; Rice et al., 2016; Yamashita et al., 2016; Giordano et al., 2018; Sotoyama et al., 2021).

The VTA is believed to be involved in reward, motivation, addiction, sleep, and manic behavior (D’Ardenne et al., 2008; Cohen et al., 2012; Lammel et al., 2012; Morales and Margolis, 2017). In recent years, VTA GABA neurons have been increasingly studied in addition to VTA DA neurons (Lee et al., 2001; Brown et al., 2012; Cohen et al., 2012; Tan et al., 2012; van Zessen et al., 2012; Bocklisch et al., 2013; Yoo et al., 2016; Yu et al., 2021; Lowes et al., 2021). Yu et al. (2021) found that dysfunction of the VTA GABA neurons can lead to manic-like behaviors in mice, while activation of these neurons can induce sedation in mice, which is partly consistent with our conclusion. Therefore, it is reasonable to speculate that DBS in the VTA can also play a therapeutic role in manic symptoms in patients with manic or bipolar disorders by activating GABA neurons in the VTA. In addition, electrical stimulation of the VTA activates VTA DA neurons (Figure 3), which can project and release dopamine to other brain regions, such as the nucleus accumbens (NAc) and the prefrontal cortex (PFC), and may also relieve depression through this mechanism, since activation of VTA DA neurons has previously been reported to have antidepressant effects (Tye et al., 2013).

Many theories have been proposed to explain the mechanism of DBS’s therapeutic effects, such as neuronal activity inhibition theory, synaptic inhibition theory, decoupling theory, and neural network functional reorganization theory (Montgomery and Gale, 2008; Miocinovic et al., 2013; Herrington et al., 2016). In our study, although electrical stimulation of the VTA could activate both VTA GABA and DA neurons (Figures 3 and Figures 4), only the activation of the VTA GABA neurons was found to underlie the behavioral effects (Figures 5 and 6). Anatomically, VTA GABA neurons not only interact with local DA neurons, but also have long and dense projections to other brain regions, such as the lateral hypothalamus, NAc, and ventral pallidum (Brown et al., 2012; Taylor et al., 2014; Breton et al., 2019). We suspect that the long projections initiated from the VTA GABA neurons facilitated the antipsychotic effects caused by electrical stimulation of the VTA, which is like a master controller of the brain and body motor activity.

In conclusion, electrical stimulation of the VTA could activate VTA GABA neurons to suppress the positive symptoms of psychosis. This preclinical study not only provides a new methodological perspective on the mechanism by which DBS exerts its therapeutic effects but also proposes a potentially novel clinical treatment for psychotic diseases by electrical stimulation of the VTA.

## Data Availability Statement

The original contributions presented in the study are included in the article, further inquiries can be directed to the corresponding author/s.

## Ethics Statement

All experiments involving mice were carried out in accordance with the US National Institutes of Health Guide for the Care and Use of Animals under an Institutional Animal Care and Use Committee approved protocol and Association for Assessment and Accreditation of Laboratory Animal Care approved Facility at the ShanghaiTech University.

## Author Contributions

JH, XZ, and CL designed the study and wrote the manuscript. CL performed the deep brain stimulation behavioral experiments, optogenetic manipulation experiments and analyzed the behavioral data. CL completed the *in vivo* fiber photometry and Ca^2+^ signal analysis, and performed the local field potential recording. YF completed the local field potential electrode implantation and completed the analysis. HL participated in setting up the equipment of DBS. ZG provided the code for processing behavioral data. All authors contributed to the article and approved the submitted version.

## Funding

This work was supported by the National Natural Science Foundation of China (grant nos. 31922029, 31671086, 61890951, and 61890950 to JH and grant no. 81701049 to XZ).
